# Assessment of the antifungal activity of *Actinidia deliciosa* derived exosome like nanoparticles

**DOI:** 10.1038/s41598-025-13792-9

**Published:** 2025-08-18

**Authors:** Dina Salem, Ahmed Z. Abdel Aziz

**Affiliations:** 1 Department of Agricultural Biotechnology, College of Biotechnology, Misr University for Science and Technology, P.O. Box 77, Giza 12566, 6th of October City, Egypt; 2 Department of Environmental Biotechnology, College of Biotechnology, Misr University for Science and Technology, P.O. Box 77, Giza 12566, 6th of October City, Egypt

**Keywords:** Exosome-like nanoparticles (ELNs), *Actinidia deliciosa*, *F. oxysporum*, *F. solani*, *B. cinerea*, LC-MS/MS, Cell biology, Organelles

## Abstract

The Exosome-like nanoparticles (ELNs) are essential for both intra- and inter-kingdom communication. The presence and function of plant ELNs discharged into the environment remain unknown, despite their significant biological significance. In the present study, polymer precipitation method was used to isolate the ELNs from *Actinidia deliciosa* followed by characterization and antifungal activity evaluation against *F. oxysporum*,* F. solani*, and *B. cinerea* in the term of germination and density of fungal spores’ formation. Root-released EVs (extracellular vesicles) were found to have a size ranged from 30 to 150 nm, according to biophysical assessments using dynamic light scattering, and transmission electron microscope. Thirty-three unique proteins were identified using LC-MS/MS of ELNs. Several of these proteins are known to be associated with interactions between plants and microbes. The presence of multiple plant defense-related proteins in these EVs raises the possibility that they are important elements of plant immunity.

**Supplementary Information:**

The online version contains supplementary material available at 10.1038/s41598-025-13792-9.

## Introduction

Exosomes are types of extracellular vesicles composed of bio-membrane carrying special types of cargo like DNA, protein, mRNA, miRNA^1^^,^^2^ in addition numerous investigations have demonstrated that exosomes include transcription factor receptors^3^^[,4^ cytokines^5^ and other bioactive substances^6^. Exosomes are involved in many functions inside the plant. They serve a crucial function in cellular communication^7–9^. involvement in pathogenesis^10–12^ and maintaining cell homeostasis^13^. Recent studies showed that plant exosomes containing many defense response proteins^14–16^. In tomato, the proteome profile of root-released exosomes revealed the presence of different types of stress-related proteins such as endo-chitinase, ethylene-responsive proteinase inhibitor, and hypersensitive-induced response proteins^17^.

In addition, exosomal lipids are also considered as an important element in plant defense response. Recent studies showed that they act as signaling molecules^18–20^ participating in plant defense to different types of stressors^21^. The composition of membrane altered in response to biotic stressors. For example, the ratio of phospholipids including phosphatidic acid (PA) and phosphatidylinositol-4-phosphate (PI4P) increased significantly in case of fungal infection^22^ Other plant lipids are also involved in signaling mechanisms like phospholipids, oxylipins, fatty acids, sterols, and sphingolipids^23^^[,24^.

Upon all of that, exosomes contain small RNAs function in silencing genes of virulence in attacking pathogen to combat their invasion. This is one of the mechanisms by which plants defend themselves against such type of biotic stressor^25,26^.

The plant ELNs not only carry proteins, small RNAs, and signaling lipids to participate in plant defense against pathogens, but also phytochemicals^10^ which elicit the plant defense response against pathogen infection^10^. Some plant Extracellular Vehicles (EVs) including ELNs are carrying defense molecules as glucosinolates (GSLs)^27^. GSLs are enriched in many plants like *Brassicaceae* plants, and their production increased in case of pathogen infections^28,29^. GSLs are turned into active compounds by myrosinases. Hence, they perform a vital role in plant resistance to pests as they are toxic to them^29^.

It was found that isolated extracellular vesicles (EVs) from Arabidopsis contain a varied collection of short RNAs (sRNAs) that target genes linked to fungal virulence. The fungus *B. cinerea* can absorb these EVs, and the sRNAs within the EVs can be detected in fungal cells isolated from infected plants. This suggests that plant extracellular vesicles facilitate the transfer of short RNAs from host plants to fungal cells, resulting in the silencing of virulence genes and enhancing plant defense systems. These mRNAs can be introduced into *B. cinerea* cells via EVs, where they can be translated to produce proteins. The incorporation of these mRNAs into *B. cinerea* has demonstrated the ability to impede pathogen infection in host plants^24,30^.

ELNs demonstrate both growth-inhibitory effects on microbial cells and the induction of microbial cell death. The study by Regente et al. (2017) demonstrated that plant-derived extracellular vesicles can be assimilated by fungal cells, resulting in significant growth abnormalities and ultimately cell death. Furthermore, EVs induce inhibition of spore germination, hinder mycelial expansion, and result in diminished vitality. Plant extracellular vesicles limit fungal growth and promote cell death by delivering tiny RNA molecules that selectively target fungal virulence genes^31^.

The plant derived ELNs were proved to be absorbed by fungal species which enable them to silence the pathogen virulence genes^24,31^. These studies proved that EVs are absorbed by fungal cells by the aid of integral membrane proteins which present on the membrane of EVs, which facilitate the ELNs absorption by fungal cells, thereby exerting significant effect in plant resistance to fungal infection^24,31^.

Much research has been performed to study the mammalian^32–34^ fungal^35^ and bacterial EVs^36^ determining their composition, nature and their function in cellular interaction. However, the plant EVs are still poorly studied. So that, this study was conducted to isolate, characterize and proteome analysis of *Actinidia deliciosa* exosome-like nanoparticles, and assess their antifungal activity against *F. oxysporum*. *F. solani*, and *B. cinerea invitro.*

## Materials and methods

### Isolation and purification of *Actinidia deliciosa* exosome-like nanoparticles

The Exosome Like Nanoparticles (ELNs) were isolated according to Kalarikkal et al. (2020)^37^. Briefly, 500 g of fresh fruit was brought from the market then washed, peeled, weighed and then mixed with a blender. The fruits were blended at medium speed for 3 min with 30 s on/off cycles. The juice was passed through cheese cloth to eliminate fruit fibers. The filtrate was then centrifuged at 2000 g for ten minutes, 6000 xg for twenty minutes and 10,000 xg for forty-five minutes. The obtained supernatant was then mixed with PEG6000. The concentration of PEG was 15%. The mixture was incubated in the refrigerator at 4 °C overnight, followed by centrifugation at 8000 g for 30 min at 4 °C in a fixed angle rotor (Eppendorf). The supernatant was discarded and the remnant liquid containing PEG removed by turning the falcon upside down for 5 min on a filter paper. The ELNs were then dispersed in water and dialyzed for 12 h against sterile milli Q grade water by employing Dialysis membrane (Himedia) with a 10 kDa pore size.

### Transmission electron microscopy (TEM)

One µg of ELNs (in 10 µL) was placed on the copper mesh, then left at room temperature (RT) for 1–2 min, then excess liquid was eliminated; 30 µL of 2% phosphotungstic acid was mixed with copper wire, then left at RT for half minute, excess dye was removed; then it was left to dry for 5–10 min; finally, it was seen and photographed with TEM (Japan, HITACHI, H-7650 type).

### Dynamic light scattering (DLS)

The size of ELN was determined by a Zetasizer Nano ZS system (Malvern Instruments, Malvern, UK) using the DLS technique analyzing the velocity distribution of particle movement. The particle’s diameter is then measured with the Stokes–Einstein equation. After ELNs isolation with Polymer based precipitation, the ELNs pellets were diluted in 100 µl sterile milli Q grade water, then 5 µl were taken and added 495 µl to them, them mixed well by vortex at medium speed for one minute. The ELNs preparation was then transferred to cuvette to determine the size of ELNs. Three replicas were measured for each sample and the average was calculated.

### Protein extraction

The total proteins were extracted from ELNs as follows, Firstly, 100 µl volume of 8 M Urea (500 mM Tris pH 8.5) were mixed with 50 µl of ELNs preparation. Then the solution was centrifuged at 10,000 rpm for half an hour. The supernatant was then taken to complete downstream operations, the bicinchoninic acid assay (BCA assay) was employed to measure protein concentration.

### Protein denaturation and digestion

After the measurement of protein concentration, the isolated protein was denatured as follows: firstly, two microliters of DTT (200mM) were mixed with protein preparation, then the solution was mixed using vortex and let at room temperature for forty-five minutes. After that, the mixture was left for forty-five minutes at room temperature in dark condition after mixing with 2 µl of 1 M Iodoacetamide. Finally, the mixture including 102 µl of 100mM Tris pH 8.5 and 6 µl Trypsin containing 1 µg porcine enzyme was incubated in shaker incubator for 12 h at 37 °C. The solution was acidified to reach the pH 2 with 6 µl of 100% Formic acid and the Eppendorf was inverted on a paper tissue for half an hour. Then, the peptide concentration was assessed by bicinchoninic acid assay.

Ten microliters containing 1 µg of peptides were injected into trap column ChromXP C18CL (5 μm (10 × 0.5 mm)). The sample eluted into a C18 analytical column 3 μm, ChromXP C18CL (120Å, 150 × 0.3 mm). The mobile phase included: A: LC-MS water containing 0.1% fluoroacetic acid, and B: Acetonitrile containing 0.1% fluoroacetic acid. The mobile phase gradient time was 57 min, as shown in Table (1).

**Table 1:** Mobile phase gradient elution composition.

Time (min)	% A	% B
0	97	3
38	70	30
43	60	40
45	20	80
48	20	80
49	97	3
57	97	3

The flow rate was 5 µl/min. Raw MS files from the TripleTOFTM 5600 + files were analyzed by Protein pilot (version 5.0.1.0, 4895), paragon Algorithm (version 5.0.1.0, 4874). Statistical significance was measured using q-values (FDR). The used Databases was Uniprot *Actinidia deliciosa* organism (Swiss-prot and TrEMBL database containing 679 protein).

### Bioinformatic analysis

Right-tailed Fisher’s exact test was used to access Gene enrichment of three ontologies (Biological processes, cell components, and molecular functions). GO (Gene Ontology) analyses were performed using ShinyGO website. In addition, protein-protein interaction was assessed using String database. All statistical analyses were conducted using unpaired t-tests. A difference was deemed significant when *P* < 0.05. Every test was run in three replicas.

### Antifungal activity test

The antifungal activity of ELNs was measured by employing the disc diffusion method. Firstly, the fungal strains *Fusarium oxysporum* radicis lycopersici, *Fusarium solani*,* and Botrytis cinerea* were brought from Microbial Resource Center (MIRCEN). The fungi were maintained on PDA medium. The spore suspension was prepared from the culture by adding fifteen ml of sterile water followed by scraping the mycelia and filtering the suspension using cheese cloth. The spore concentration was determined using hemocytometer. The spore suspension was diluted to reach 10^6^ spore/ml. The suspension was spread on PDA media to assess the antifungal activity, sterile commercial discs, with a diameter of 6.0 mm, were infused with various dilutions of the isolated exosomes, ranging from 125 to 500 µg/ml. The zones of inhibition (ZI) have been expressed in millimeters.

### Effect of ELNs on spore density

The spores of *F. solani*,* F. oxysporum*, and *B. cinerea* were obtained after adding sterile distilled water to a culture kept in a PDA slant-tube. The spores were collected by sieving the suspension through filter paper (Whatman No. 2). 600 µl of PDB were placed in Eppendorf tubes and mixed with the three concentrations of ELNs (125, 250 and 500 µg/ml). A volume of 100 µl spore suspension was added with the final concentration of 10^6^ spores/ml). Finally the total volume was completed to 1 ml with PDB. The cultures were incubated at 28 ± 2 °C in the dark for five days. Using hemocytometer, the spore number was determined by employing corresponding equation^38^

$${\rm{IS}} = (dc - dt/dc)*100$$

*IS*: The spore formation suppression, *dc*: control spore, and *dt*: The spore density after applying ELNs.

### Effect of ELNs on spore germination

To test the effect of treatments on spore germination of the three phytopathogens, 600 µl of PDB were placed in Eppendorf tube and mixed with the three concentrations of ELNs (125, 250 and 500 µg/ml). A volume of 100 µl spore suspension was added with the final concentration of 10^6^. The total volume was completed to 1 ml. For every concentration, three Eppendorf tubes were prepared and left for a duration of 12 h in the dark at room temperature (28 ± 2 °C). The number of germinated spores was determined by employing a hemocytometer^38^.

### Statistical analysis

One-way analysis of variance (ANOVA) was used allowing for the investigation of differences between the means of the various groups. The results were represented as means ± standard error of the mean (SEM), *P* < 0.05 was used to evaluate significance of the results.

## Results

### ELNs preparation and characterization

**Fig. 1:**
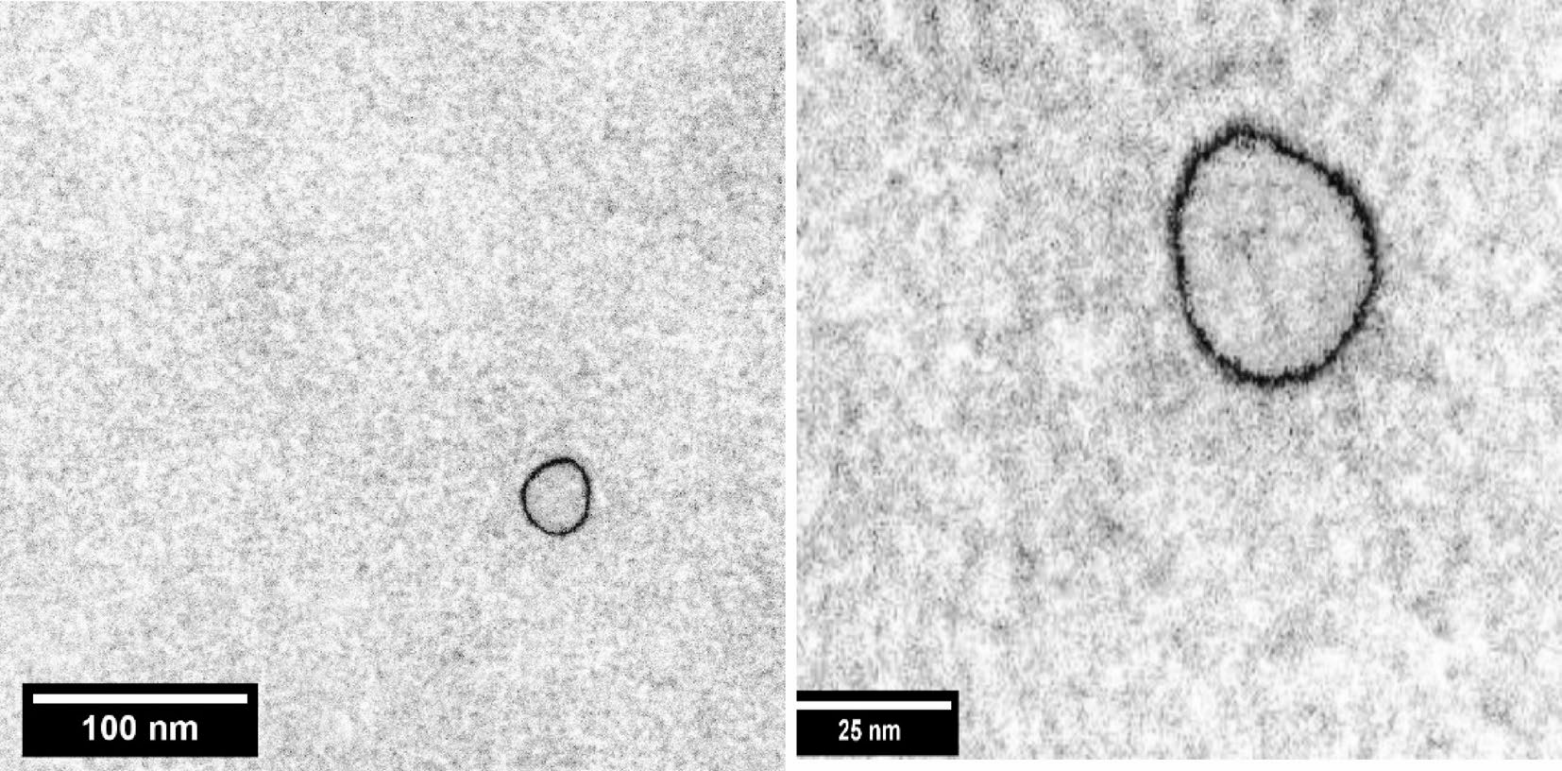
TEM image of the *A. deliciosa* exosomes.

**Fig. 2:**
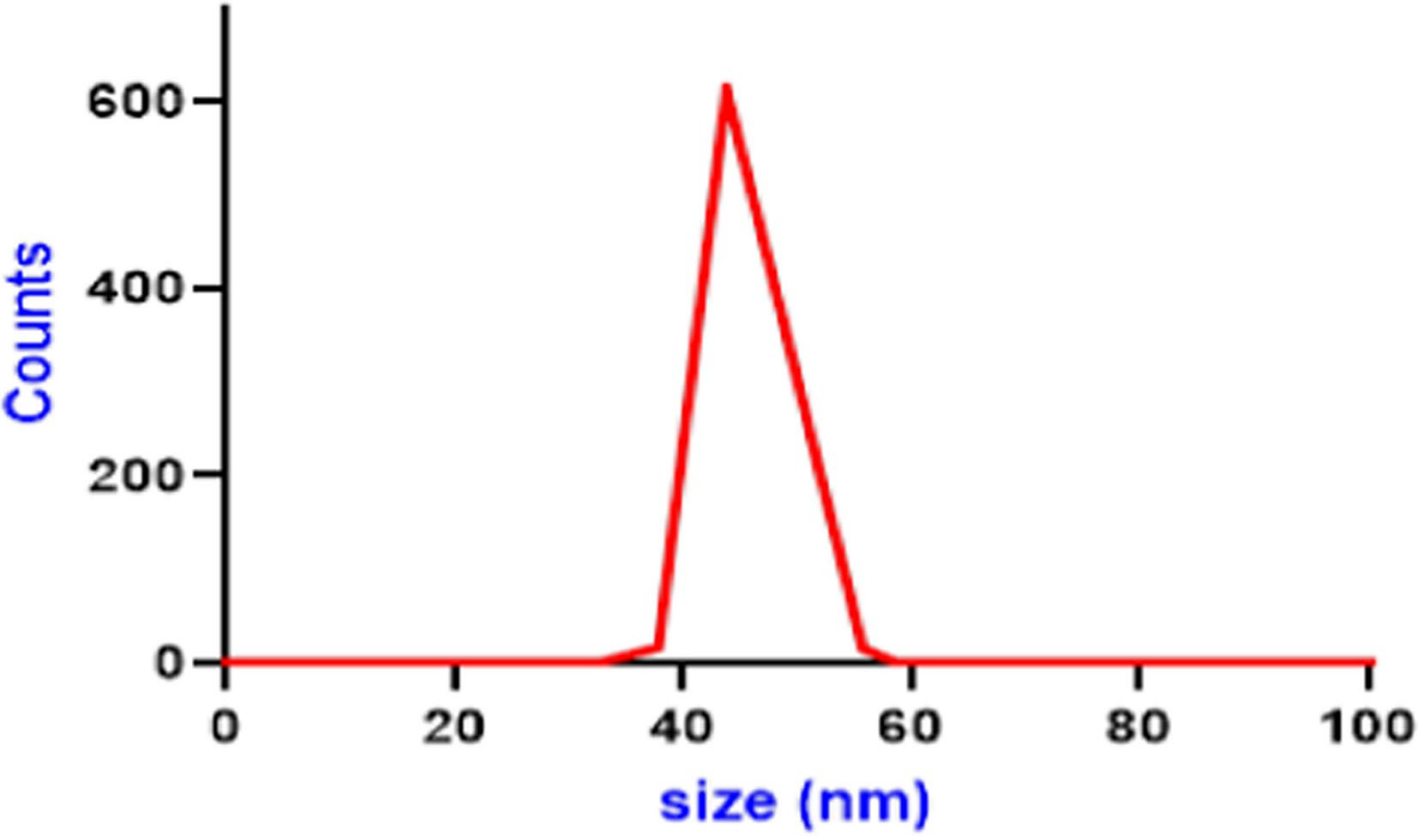
The DLS of *Actinidia deliciosa* extracellular vesicles.

The TEM characterization of extracellular vesicles (Exosome-like nanoparticles) showed that the average size of *Actinidia deliciosa* extracellular vesicles (exosome) is 35.6 ± 1.16 nm. TEM images indicate that EVs are spherical to oval shaped (Fig. 1). The results of Dynamic Light Scattering (DLS) analysis of the *A. deliciosa* extracellular vesicles (exosome) showed narrow size distribution, the size ranged from 38 to 60 nm with average size 43 nm as shown in the Fig. (2).

###  Proteome analysis of ELNs

**Fig. 3:**
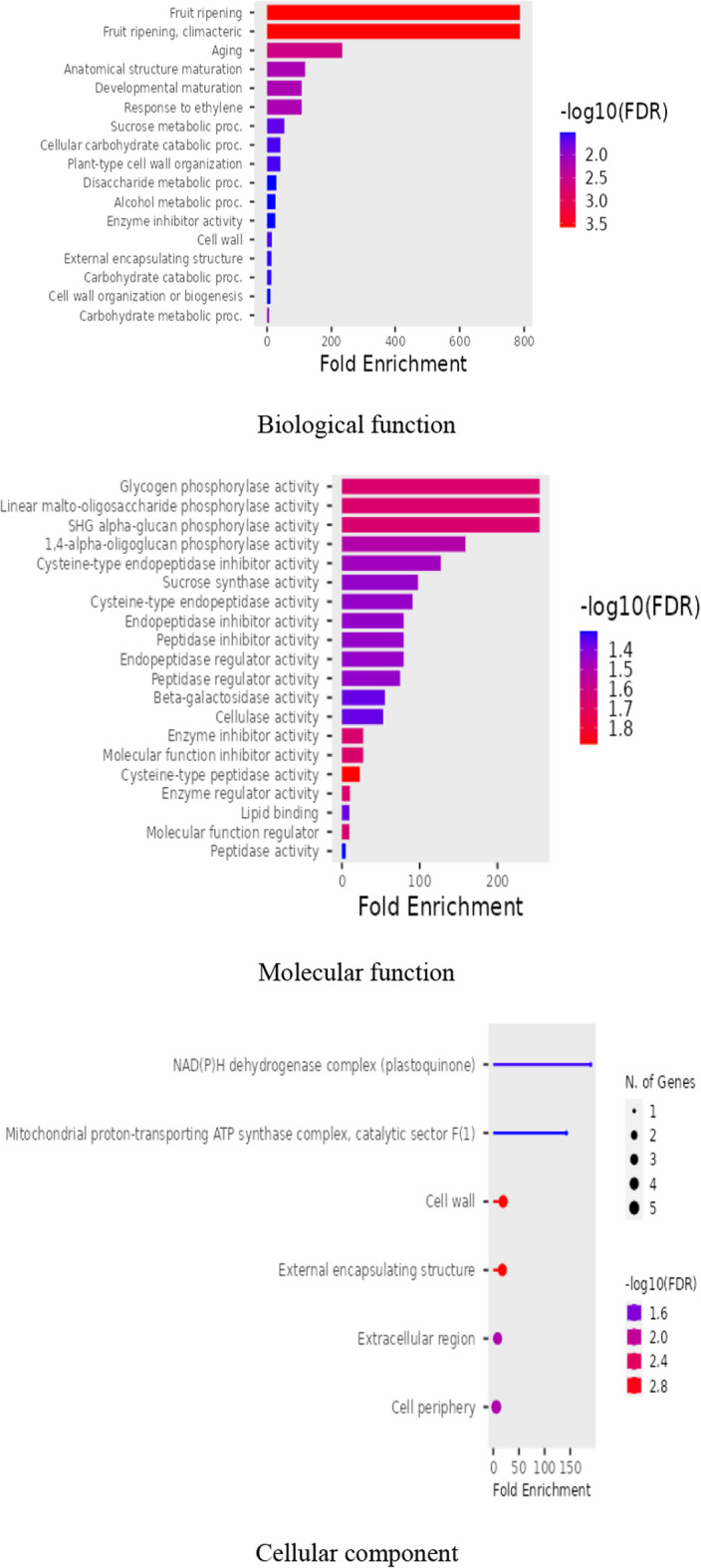
The GO analysis of *A. deliciosa* Exosome-Like Nanoparticles.

The proteome analysis revealed the presence of 32 unique proteins. Many of the isolated proteins have been detected in other reports in extracellular vesicles. The gene ontologies of these proteins in terms of Biological Process were fruit ripening, aging, cell wall organization, metabolic processes, external encapsulating structure, response to ethylene, development, cell wall organization, external encapsulating structure, and carbohydrate metabolism; while in terms of their molecular functions included Cysteine-type peptidase activity, Enzyme inhibitor activity, “Enzyme regulator activity, Linear malto-oligosaccharide phosphorylase activity, Molecular function inhibitor activity, Cysteine-type endopeptidase inhibitor activity, Endopeptidase inhibitor activity, Peptidase inhibitor activity, Endopeptidase regulator activity, Lipid binding, and hydrolase activity. Additionally, The Gene Ontology (GO) analysis in terms of cellular components revealed that the isolated proteins belong to external encapsulating structure, cell periphery, mitochondrial proton transport- ATP synthase complex, cell wall, and Plastoquinone (Fig. 3).

**Fig. 4:**
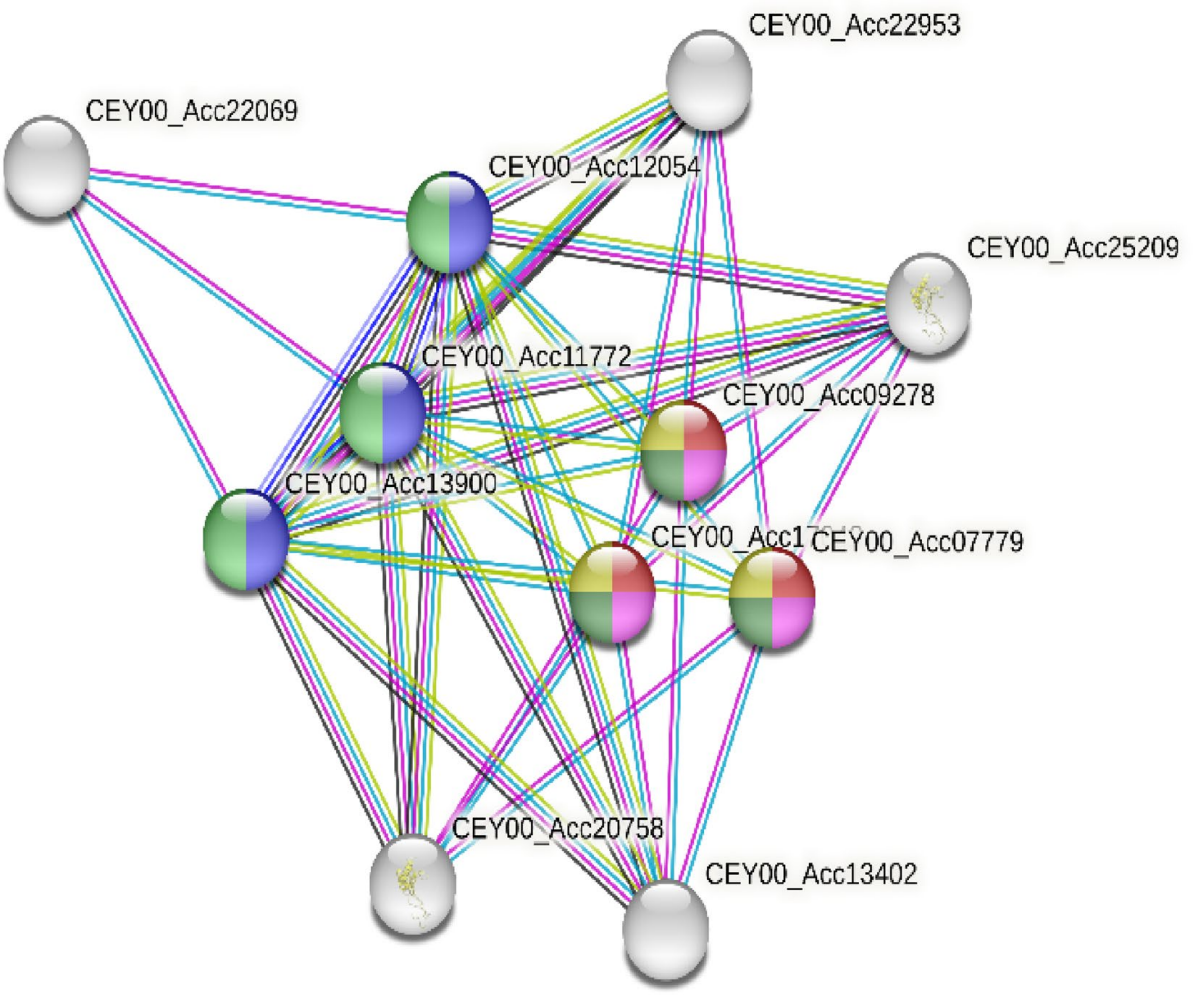
The protein-protein interaction between *A. deliciosa* ELNs identified protein.

The protein–protein interaction (PPI) network of identified proteins was represented in Fig. (4) by employing STRING database. It was worth mentioned that the exosomal proteins which identified at central nodes as possible candidate biomarkers including phosphobilinogen deaminase, Actinidain-like and Vignain-like, which belong to the peptidase C1 family, 40 S ribosomal protein-like, which is a member of uS11 universal ribosomal protein family, Protein transport protein Sect. 31 B-like and UPF0235 protein.

The red color refers to proteins involved in COPII-coated vesicle cargo loading, blue color refers to proteins involved in Extracellular space, green color phagosome pathway, yellow color refers to ER to Golgi Anterograde Transport and mint green refers to COPII-coated vesicle budding.

The process of ER-derived vesicles, which carry newly generated proteins to the Golgi compartment, is initiated by coat protein complex II (COPII). One of this protein vital functions is to facilitate proteins’ extracellular transit and localization to different intracellular compartments like Golgi apparatus.

### Antifungal evaluation of ELNs

The results obtained from the disc-diffusion method showed absence of any inhibition zone with all of the tested three fungi.

### Effect of *A. deliciosa* ELNs onfungal spore density and germination

The statistical analysis showed that treatment of the phytopathogenic fungi spores with the applied concentrations of ELNs had significantly decreased the density and germination of *F. oxysporum* and *(A) solani*, and *(B) cinerea* spores (Table 2).

The highest decrease in spore density was recorded after treating *F. solani.* spores with the concentration of 500 µg/ml of ELNs, which decreased the density by 75.53%, as compared with control. On the other hand, the highest decrease in spore germination was recorded after treating spore suspension of *F. oxysporum* with the concentration of 500 µg/ml of the ELNs, which decreased the spore germination by 66.79% as compared with control.

**Table 2:** Effects of ELNs on density, and germination of *Botrytis cinerea*,* Fusarium oxysporum*, *Fusarium solani *spores.

	Botrytis cinerea		Fusarium oxysporum		Fusarium solani	
Conc**.** (µg/ml)	Spore density Spore/ml ×10^6^	Spore germination Spore/ml ×10^6^	Spore density Spore/ml ×10^6^	Spore germination Spore/ml ×10^6^	Spore density Spore/ml ×10^6^	Spore germination Spore/ml ×10^6^
Control	8.6^a^ ± 0.2	1.48^a^ ± 0.21	10.43^a^ ± 1.23	2.65^a^ ± 0.60	6.5^a^ ± 1.1	1.77^a^ ± 0.61
125	8.36^a^ ± 0.088	1.35^ab^ ± 0.34	7.57^b^ ± 0.34	1.24^a^ ± 0.54	4.7^a^ ± 0.26	1.44^ab^ ± 0.42
250	6.2^b^ ± 0.15	1.27^abc^ ± 0.29	5.07^b^ ± 0.55	0.87^b^ ± 0.29	2.73^b^ ± 0.82	0.95^abc^ ± 0.48
500	5.3^c^ ± 0.057	0.8^abcd^ ± 0.17	3.8^c^ ± 0.85	0.88^b^ ± 0.41	1.59^b^ ± 0.41	0.72^abcd^ ± 0.24

## Discussion

Exosomes perform a crucial function in the interaction between the cells within the same organism. They have the ability to transport a broad variety of substances, including lipids, proteins, RNA, and DNA, which control different pathways in recipient cells located at specific locations^39^, also they are involved in interaction between host and pathogen^40^. To comprehend the role of extracellular vesicles in such interaction the proteomic analysis was done to identify different types of proteins present in ELNs and their role in combating pathogen attack.

The identified proteins were categorized by GO analysis into biological function, molecular function, and cellular component. According to biological function the identified proteins were involved in functions like fruit ripening, aging, cell wall organization, metabolic processes, external encapsulating structure, response to ethylene, development, cell wall organization, external encapsulating structure, and carbohydrate metabolism. According to their molecular functions they are divided into Cysteine-type peptidase activity, Enzyme inhibitor activity, “Enzyme regulator activity, Linear malto-oligosaccharide phosphorylase activity, Molecular function inhibitor activity, Cysteine-type endopeptidase inhibitor activity, Endopeptidase inhibitor activity, Peptidase inhibitor activity, Endopeptidase regulator activity, Lipid binding, and hydrolase activity. While they were categorized to external encapsulating structure, cell periphery, mitochondrial proton transport- ATP synthase complex, cell wall, Plastoquinone according to the cellular components to which they belong.

Some of the identified proteins involved in defense response against pathogens such as; Kiwellin^41^ thaumatin-like protein^42^, pectin esterase inhibitor^43^, Polygalacturonase inhibitor^44^ WRKY1, and WRKY2 transcription factors^45^ acidic chitinases, and β-(1,3)-glucanases. The presence of plant defense response proteins in EVs was also reported by other studies, where it was a common denominator between all the plant derived EVs.

Cell wall remodeling enzymes (CWREs) are also from the commonly isolated proteins from plant EVs. The current study showed the presence of different CWREs like expansin^46^ pectinesterase inhibitor^47^. Expansins, are generated by the golgi apparatus, these enzymes secreted outside of the cell for modifying cell walls to promote plant cell size increase^48^. Moreover, pectinesterase inhibitor, another enzyme that remodels cell walls and aids in plant defense against infections^49^.

Furthermore, LC-MS/MS recorded the presence of antifungal proteins like chitinase, thaumatin-like proteins, and endoglucanase, these types proteins (secreted outside the cell) were also isolated from extracellular vesicles in other studies^50^.

Lipid transfer protein transport phospholipids and galactolipids through cellular membranes^51,52^. They participate in wax or cutin accumulation rtain secretory tissues^53^. These types of proteins had been identified previously in Arabidopsis extracellular vesicles^48^.

Also, from the proteins isolated in the current study and in other EVs, Polygalacturonase inhibitor protein is one of defense proteins, it has a considerable role in plant resistance to phytopathogenic fungi, acting as an *Inhibitor* of fungal *polygalacturonase*^54^. Also, Endoglucanase: have been recorded in xylem and phloem exosomes which involved in transferring endo-1,4-β-glucanases^55^. Pectin esterase and pectin esterase inhibitor are Cell wall related proteins. They have been recognized in previous studies^56^.

α−1,4-glucan phosphorylase (glycogen phosphorylase family) recorded in different researches in mammalian extracellular vesicles, its one of the glycosyl hydrolase which had been identified in extracellular vesicles of many organisms like protozoa *Acanthamoeba castellanii*^57^.

The glycosyl hydrolase enzyme invertase has also been found in the extracellular vesicles of mammalian cells, and it is thought to be a marker of secretory vesicles in yeast^58^. In plants, Extracellular invertase up-regulation seems to be a typical response to certain stress-related reactions as well as a range of both biological and environmental stress-related triggers, such as pathogen infection and salt stress^59^. This protein also had been identified in watermelon extracellular vesicles^60^.

Recently, ATP synthase has been found in several extracellular vehicles (EVs), which are crucial for cellular interaction, according to many investigations that used proteomics techniques. Nevertheless, it is still unknown what roles ATP synthase plays in EVs. It was discovered in FYN, a kinase associated with T cell receptor signaling that is present at the plasma membrane of Jurkat T cells. It was proposed that FYN may have an effect on Jurkat T cell proliferation via interacting with eATP synthase in exosomes^61^.

Plant defense proteins are among the primary protein types found in exosomes; several proteins found in extracellular vesicles (EV) have been identified as pathogenesis-related (PR) proteins^62^. These proteins include chitinases II (PR-4), thaumatins (PR-5), proteinase inhibitors (PR-6), peroxidases (PR-9), and lipid transfer proteins (PR-14). chitinases II (PR-4), thaumatins (PR-5), proteinase inhibitors also were identified in the present study^31^.

Thaumatin protein is one of the identified proteins of the current study, which proved to exert antifungal activity by altering the permeability of the plasma membrane, hence dissipating the pH gradient^63^, or resulting in the leaking of cytoplasmic contents^64^. Instead, these proteins attach to particular cell membrane receptors in *Saccharomyces cerevisiae*, triggering cellular death through a RAS2 signaling pathway^65^.

Nonspecific lipid-transfer proteins are also among the identified antifungal proteins. The examination of antifungal activity indicated that these nonspecific lipid-transfer proteins could impede growth by interacting with the fungal membrane^66^.

Bolygalacturonase-blocking proteins (PGIPs) are crucial for combating fungal infections by inhibiting the pectin-depolymerizing activity of endopolygalacturonases (PGs), a category of enzymes released by pathogens that weaken plant cell walls and increase susceptibility to illness^67^. Cysteine proteinase demonstrated considerable antifungal effectiveness against pathogenic fungi, which is associated with inhibiting the formation of the fungal cell wall by interfering with chitin synthesis^68^.

The pectinesterase inhibitor in Arabidopsis was demonstrated to enhance the degree of pectin methyl esterification in the pectin cell wall, hence diminishing the plant’s vulnerability to fungal and bacterial necrotrophs^69,70^. Furthermore, the resistance of plants to fungal infections is diminished in Pectine methyl esterase inhibitor mutants with decreased PMEI expression^71^. In addition, GhPMEI3 (pectin methylesterase-inhibiting protein GhPMEI3 from cotton) effectively inhibited fungal mycelial growth. Therefore, it may serve to strengthen the cell wall barrier and enhance antifungal resistance in cotton.

Kiwellin, a secreted protein found outside the cell membrane, belongs to a large family of plant resistance-related proteins that specifically attack certain microbe effectors. For example, the activity of the secreted chorismate mutase Cmu1 enzyme, the virulence related protein of *Ustilago maydis*, is selectively inhibited by the KWL1 protein produced by its infected corn^72,73^.

The results showed that *A. deliciosa* ELNs exerted significant antifungal activity on *F. oxysporum*, and *F. solani* in spore density and spore germination assays, while showing no inhibitory zones in disc diffusion method, which indicated that ELNs have fungistatic, but not fungicidal properties. The antifungal properties of exosomes were previously recorded in tomato exosomes against *F. oxysporum*, *Alternaria sp*,* and Botrytis cinereal* spore germination. Also, the oral mucosa exosomes showed antifungal activity against Candida. The antifungal activity of *A. deliciosa* ELNs is attributed to presence of different defense related proteins like Kiwellin, thaumatin-like proteins, pectiesterase inhibitor. Kirola, Polygalacturonase inhibitor, WRKY1, WRKY2 transcription factors, in addition *A. deliciosa* ELNs may contain phytochemicals with antifungal activity like gallic acid, this needed to be confirmed by analysis of ELNs phytochemical composition. In addition, Cai et al. 2019 found plant EVs help transfer small RNAs (sRNAs) from the infected plant to pathogen, which in turn silences virulence genes and strengthens plant defense mechanisms^25^.

## Conclusion

Extracellular vesicles perform many functions in living organisms, helping in cell communication, waste management, and involved in host pathogen interaction. Many studies have been done on mammalian EVs, However, the plant EVs are poorly studied. ELNs secreted by *A. deliciosa* contain homologs of proteins that are normally recorded in extracellular vesicles derived from mammals. The discovery that ELNs of *(A) deliciosa* contain many plant defense proteins without infections raises the possibility that EVs are included in a physiological aspect of the immune system of plants. In fact, we demonstrated their in vitro fungal infections against three plant pathogens including *(B) cinerea*,* F oxysporum*,* and F. solani*, and ELNs of noninfected A deliciosa exerted significant antifungal activity against three pathogens in terms of the suppression of spore density and spore germination. More studies needed to be done to understand the function of plant ELNs in the interaction between plants and their infectious agents.

## Supplementary Information

Below is the link to the electronic supplementary material.

Supplementary Material 1

## Data Availability

Data is provided in supplementary information files.
